# Association of Neutrophil to Lymphocyte Ratio With Pulmonary Function in a 30-Year Longitudinal Study of US Veterans

**DOI:** 10.1001/jamanetworkopen.2020.10350

**Published:** 2020-07-13

**Authors:** Xu Gao, Brent Coull, Xihong Lin, Pantel Vokonas, David Sparrow, Lifang Hou, Dawn L. DeMeo, Augusto A. Litonjua, Joel Schwartz, Andrea A. Baccarelli

**Affiliations:** 1 Laboratory of Environmental Precision Biosciences, Mailman School of Public Health, Columbia University, New York, New York; 2 Department of Biostatistics, Harvard T.H. Chan School of Public Health, Boston, Massachusetts; 3 Veterans Affairs Normative Aging Study, Veterans Affairs Boston Healthcare System, Boston, Massachusetts; 4 Department of Medicine, Boston University School of Medicine, Boston, Massachusetts; 5 Department of Preventive Medicine, Feinberg School of Medicine, Northwestern University, Chicago, Illinois; 6 Channing Division of Network Medicine, Brigham and Women’s Hospital, Boston, Massachusetts; 7 Division of Pulmonary and Critical Care Medicine, Brigham and Women’s Hospital, Boston, Massachusetts; 8 Division of Pediatric Pulmonary Medicine, Golisano Children's Hospital at Strong, University of Rochester Medical Center, Rochester, New York; 9 Department of Environmental Health, Harvard T.H. Chan School of Public Health, Boston, Massachusetts

## Abstract

**Importance:**

Chronic obstructive pulmonary disease (COPD) is a critical public health burden. The neutrophil to lymphocyte ratio (NLR), an inflammation biomarker, has been associated with COPD morbidity and mortality; however, its associations with lung function decline and COPD development are poorly understood.

**Objective:**

To explore the associations of NLR with lung function decline and COPD risks.

**Design, Setting, and Participants:**

This longitudinal cohort study included white male veterans in the US with more than 30 years of follow-up to investigate the associations of NLR with lung function, COPD, and hypomethylation of cg05575921, the top DNA methylation marker of lung function changes in response to tobacco smoking. This study included 7466 visits from 1549 participants, each examined up to 13 times between 1982 and 2018. A subgroup of 1411 participants without COPD at baseline were selected to analyze the association of NLR with incident COPD. Data were analyzed from September 2019 to January 2020.

**Exposures:**

The primary exposure was NLR, which was estimated using automated whole blood cell counts based on a blood sample collected at each visit. The methylation level of cg05575921 was measured in blood DNA from a subgroup of 1228 visits.

**Main Outcomes and Measures:**

The outcomes of interest were lung function, measured as forced respiratory volume in the first second (FEV_1_) in liters, forced vital capacity (FVC) in liters, percentage of FVC exhaled in the first second (FEV_1_/FVC), and maximal midexpiratory flow rate (MMEF) in liters per minute and COPD status, defined as meeting the Global Initiative for Chronic Obstructive Lung Diseases stage II (or higher) criteria. Both outcomes were measured as each visit.

**Results:**

Among 1549 included men (mean [SD] age, 68.3 [9.3] years) with 7466 visits from 1982 to 2018, a 1-unit increase in NLR was associated with statistically significant mean (SE) decreases of 0.021 (0.004) L in FEV_1_, 0.016 (0.005) L in FVC, 0.290% (0.005) L in FVC, 0.290% (0.065%) in FEV_1_/FVC, and 3.65 (0.916) L/min MMEF. Changes in NLR up to approximately 10 years were associated with corresponding longitudinal changes in lung function. Furthermore, this increase in NLR was associated with 9% higher odds of COPD (odds ratio, 1.09 [95% CI, 1.03-1.15]) for all visits and 27% higher risk of incident COPD (odds ratio, 1.07 [95% CI, 1.07-1.51]) for participants without COPD at baseline. Additionally, a 1-unit increase in NLR was associated with a mean (SE) decrease of 0.0048 (0.0021 in cg05575921 hypomethylation, which may mediate the adverse association of NLR-related inflammation on lung function.

**Conclusions and Relevance:**

These findings suggest that NLR may be a clinically relevant biomarker associated with high risk of lung function impairment and COPD alone or in combination with DNA methylation profiles.

## Key Points

**Question:**

Is the neutrophil to lymphocyte ratio (NLR) associated with lung function decline and chronic obstructive pulmonary disease risk at the population level?

**Findings:**

This cohort study of 1549 US veterans with more than 30 years of follow-up found that higher NLR was associated with lower lung function and higher chronic obstructive pulmonary disease risk. Additionally, NLR was associated with *AHRR* hypomethylation, the top DNA methylation signature of lung function changes, which may mediate the adverse association of NLR-related inflammation with lung function.

**Meaning:**

These findings suggest that NLR may be a useful, readily obtainable, and inexpensive tool to identify people with high risks of lung function impairment and chronic obstructive pulmonary disease.

## Teaser

This cohort study examines the associations of neutrophil to lymphocyte ratio with lung function and risk of chronic obstructive pulmonary disease among US veterans with up to 30 years of follow-up.

## Introduction

Tobacco smoking and abnormal progression of age-related lung function decline are underlying determinants of chronic obstructive pulmonary disease (COPD), a critical public health burden with high health care costs due to frequent hospitalization, loss of productivity, and disability.^1^ Chronic inflammation resulting from exogenous exposures perpetuates decline in lung function (eg, forced expiratory volume in 1 second [FEV_1_] and forced vital capacity [FVC]).^2,3,4^ Inflammation can be rapidly, affordably, and easily assessed through routine blood count analysis of a key biomarker: neutrophil to lymphocyte ratio (NLR),^5,6^ which correlates with clinical and functional parameters in patients with COPD and thus is a putative marker associated with COPD severity and relevant mortality.^5,7,8,9,10^ However, little is known about the associations of NLR with lung function decline and COPD development in the general population.

Smoking is the most important exogenous hazard impairing lung function.^1^ Hypomethylation in the aryl hydrocarbon receptor repressor (*AHRR*; OMIM 606517) gene, especially locus cg05595921, may be a better identifier of smoking exposure than self-reported smoking status.^11,12,13^ Methylation of cg05575921 also has a robust association with lung function decline in different populations and likely clinical utility for identifying individuals with poor lung function and accelerated decline.^13,14^ This locus may also be related to mechanisms of lung function impairment through a chronic inflammatory response related to smoking.^15^ Nevertheless, to our knowledge, no population-level studies have evaluated whether this emerging epigenetic biomarker is independent of other biomarkers possibly associated with lung function decline, such as NLR.

We sought to fill these research gaps by investigating associations of NLR with lung function and risk of COPD in a longitudinal study with more than 30 years of follow-up within the Normative Aging Study (NAS), an all-male cohort of older veterans living in the Greater Boston, Massachusetts, area. We also evaluated the associations of NLR with longitudinal changes of lung function parameters and the incidence of COPD and assessed to what extent NLR and cg0557592 were associated with lung function and COPD risks.

## Methods

### Study Design and Population

This study was approved by the institutional review boards of Columbia University and other relevant institutions. Each NAS participant provided written informed consent before participation. This report followed the Strengthening the Reporting of Observational Studies in Epidemiology (STROBE) reporting guideline.

The NAS is an ongoing longitudinal study on aging established in 1963. Briefly, the NAS is a cohort of male veterans. All participants had no known chronic medical conditions at the initial health screening and are re-examined with detailed on-site physical examinations and questionnaires conducted every 3 to 5 years on a continuous rolling basis. Owing to the small proportion of participants in NAS who are not white and because NLR distributions are race-specific,^16^ only white participants were analyzed in this study. To avoid the confounding effect of acute infection on NLR, visits during which high levels of C-reactive protein (ie, >1.00 mg/dL; to convert to milligrams per liter, multiply by 10) were detected were excluded. Participants had up to 13 visits between 1984 and 2018. Blood DNA methylation profiles were examined between 1999 and 2013 from a subgroup of randomly selected participants.

### Data Collection

Full details on data collection are provided in the eAppendix in the Supplement. Standard methods were used to obtain FEV_1_ (liters), FVC (liters), proportion of FVC exhaled in the first second (FEV_1_/FVC), and maximal midexpiratory flow rate (MMEF; liters per minute) at each of up to 13 visits between 1984 and 2018.^17^ Additionally, COPD status was defined at each visit as meeting the Global Initiative for Chronic Obstructive Lung Diseases stage II or higher criteria (ie, FEV_1_/FVC <70% and FEV_1 _<80% predicted).^18^ Smoking history, including status and pack-years, were assessed at the time of spirometry tests using the American Thoracic Society questionnaire.^19^ At each visit, NLR was calculated by dividing total absolute neutrophil counts by total absolute lymphocyte counts as a continuous variable. To explore potential cutoff points, 2 NLR cutoffs were explored as potential reference values: in category 1, an NLR of 3.00 or less was considered low, and in category 2, an NLR of 2.27 or less was considered low.^20^ The methylation level of cg05575921 was retrieved from the whole epigenome data, which were measured with the 450K BeadChip (Illumina).^21^

### Statistical Analysis

We first evaluated the cross-sectional associations of NLR with the 4 lung function parameters obtained at the same visit (linear regression) and the risk of COPD (logistic regression). We used random participant-specific intercepts to account for the correlation of repeated measures. We applied 3 models that increasingly adjusted for potential covariates obtained at the same visit. Model 1 adjusted for age, BMI (categorized as underweight or weight within reference range, overweight, and obese), and height. Model 2 additionally adjusted for smoking status (categorized as current, former, and never smoker), pack-years, alcohol consumption (categorized as abstainer or low, intermediate, and high consumption), and education (categorized as ≤12 years, 13-16 years, and >16 years). Model 3 (ie, the fully-adjusted model) additionally adjusted for hypertension, stroke, coronary heart disease, diabetes, and other chronic lung conditions. Dose-response curves and the linearity of NLR’s associations with lung function and COPD odds were assessed by restricted cubic spline regression.^22^ The 5th, 50th, and 95th percentiles were selected as knots for NLR.

Next, we separately selected participants with at least 2, 3, 4, and 5 visits to evaluate the longitudinal associations of NLR with lung function. We assessed the associations of NLR at the first visit and changes, defined as the difference between the measures of the last visit and the first visit, with corresponding lung function parameters. Change rates per year were calculated by dividing a measured change with the corresponding time range.

For time-to-event analysis of incident COPD and NLR, a subgroup of participants who did not have COPD at baseline were selected. Lacking accurate COPD incident dates, we estimated incident date as the median date between first diagnosis of COPD and the prior visit. Analysis was performed using multivariate Cox regression models with covariates obtained at the initial visit. Dose-response curve and linearity were also assessed with restricted cubic spline regression.

In the subgroup with methylation data, we tested the associations of NLR with cg05575921 methylation levels measured at the same visit. Mixed linear regression models were used to control for leukocyte distribution estimated by Houseman’s algorithm^23^ and the random batch effect of DNA methylation measurement. We also fit models including both indicators as independent variables to assess independent associations of lung function with COPD odds. We additionally explored whether the associations of NLR-related inflammation response with lung function were mediated by cg05575921 hypomethylation, or whether the converse was plausible, using mediation analysis. All the aforementioned tests were also performed in subgroups stratified by smoking status (ie never vs ever smoking) as sensitivity analyses.

In addition, as healthier study participants may be more likely to participate in subsequent clinical examinations over time, we used inverse probability weighting to correct for potential survival bias as a sensitivity analysis to evaluate the validity of the missing at random assumption and assess the effect of potential selection bias caused by nonrandom unavailability for follow-up. Weighted models that simultaneously adjusted for the inverse probability and the previously introduced covariates were conducted to validate the robustness of our primary findings, except the findings of mediation analyses.

Data cleaning and all analyses were performed using SAS statistical software version 9.4 TS1M5 (SAS Institute). A 2-sided *P* < .05 was considered statistically significant. Data were analyzed from September 2019 to January 2020.

## Results

### Participant Characteristics

A total of 7466 visits from 1549 men were eligible for the primary analysis, including 1228 visits from 708 participants in the DNA methylation subgroup. Participants in the all visits group were younger than those in DNA methylation subgroup (mean [SD] age, 68.3 [9.3] years vs 74.5 [7.1] years) (eTable 1 in the Supplement). As the DNA methylation measurements were obtained approximately 15 years after study initiation, this age difference was expected. The mean (SD) FEV_1_ of the all visits group was slightly higher than that of the DNA methylation subgroup (2.67 [0.61] L vs 2.53 [0.58] L). Similarly, the mean (SD) FVC was higher in the all visits group than in the DNA methylation subgroup (3.56 [0.70] L vs 3.39 [0.68] L). The all visits group had a lower mean (SD) NLR (2.27 [1.26]) than the DNA methylation subgroup (2.82 [1.38]). Among the all visits group and DNA methylation subgroup, more than 60% of visits were from former smokers (all visits: 4546 visits [60.9%]; DNA methylation: 797 visits [64.9%]), and approximately one-third of visits were from never smokers (all visits: 2316 visits [31.0%]; DNA methylation: 389 visits [31.7%]). Participants from the all visits group, compared with those in the DNA methylation subgroup, had a lower prevalence of hypertension (4539 visits [60.8%] vs 908 visits [73.9%]) and other chronic lung conditions (897 visits [12.0%] vs 157 visits [12.8%]). Both groups had a similar proportion of visits from patients with COPD (all visits: 765 visits [10.3%]; DNA methylation subgroup: 132 visits [10.8%]). Of 1411 participants without COPD at the initial visit, 152 participants (10.8%) were diagnosed with COPD based on the Global Initiative for Chronic Obstructive Lung Diseases criteria during a median (interquartile range) follow-up of 11.9 (14.9) years.

### Associations of NLR With Lung Function and Odds of COPD

**Figure 1.**
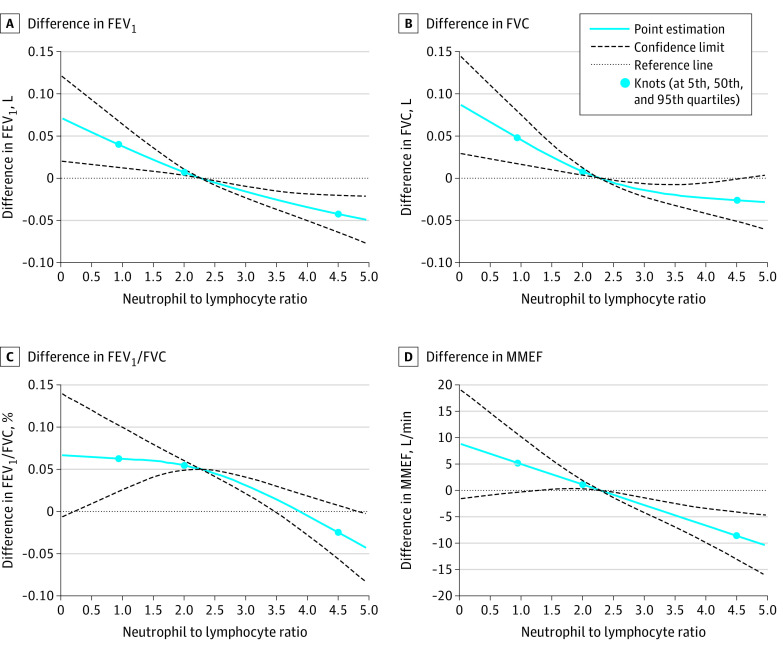
Best-Fitting Models for Relationships of Neutrophil to Lymphocyte Ratio With Lung Function Parameters FEV_1_ indicates forced respiratory volume in the first second; FEV_1_/FVC, percentage of vital capacity exhaled in the first second; FVC, forced vital capacity; and MMEF, maximal mid-expiratory flow rate.

**Figure 2.**
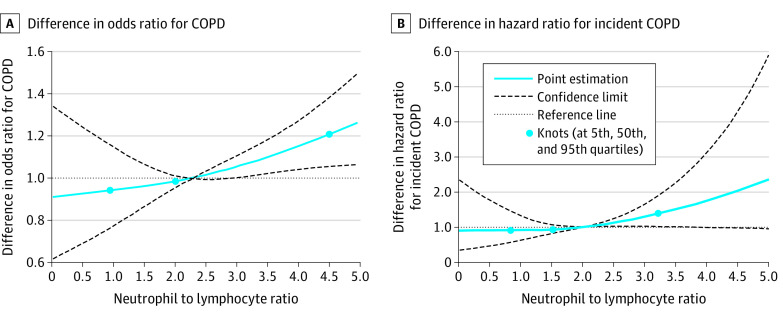
Best-Fitting Model for the Relationship of Neutrophil to Lymphocyte Ratio With the Chronic Obstructive Pulmonary Disease (COPD) Odds Ratio and the Incidence of COPD

Our analysis found that NLR was significantly associated with lower lung function parameters (Table 1). After fully adjusting for potential covariates, a 1-unit increase in NLR was significantly associated with mean (SE) decreases of 0.021 (0.004) L in FEV_1_, 0.016 (0.005) L in FVC, 0.290% (0.065%) in FEV_1_/FVC, and 3.865 (0.916) L/min MMEF. The same increase in NLR was also associated with 9% higher odds of COPD (odds ratio [OR], 1.09 [95% CI, 1.03-1.15]; *P* = .006). Monotonic negative dose-response associations of the 4 lung function parameters and COPD odds were further observed (Figure 1 and Figure 2A). For the NLR category 1 (cutoff value ≤3.00), the high NLR group demonstrated 49% higher odds of COPD compared with the low NLR group (OR, 1.49 [95% CI, 1.23-1.80]; *P* < .001) (Table 1). For NLR category 2 (cutoff value ≤2.27), the high NLR group demonstrated 27% higher odds of COPD (OR, 1.27 [95% CI, 1.07-1.51]; *P* = .005). In the sensitivity analysis by smoking status (ie, never vs ever smoking), NLR remained negatively associated with lung function parameters and positively associated with the odds of COPD in both subgroups, especially in ever smokers (eTable 2 in the Supplement).

**Table 1.** Associations of Neutrophil to Lymphocyte Ratio With Lung Function and Odds of COPD in Normative Aging Study^a^

Parameter	Patients, No. with COPD/total No.	Model 1^b^		Model 2^c^		Model 3^d^	
		Measure	*P* value	Measure	*P* value	Measure	*P* value
**Lung function, coefficients (SE)**							
FEV_1_, L	NA	−0.030 (0.005)	<.001	−0.026 (0.005)	<.001	−0.021 (0.004)	<.001
FVC, L	NA	−0.025 (0.005)	<.001	−0.022 (0.005)	<.001	−0.016 (0.005)	.001
FEV_1_/FVC, %	NA	−0.357 (0.070)	<.001	−0.316 (0.066)	<.001	−0.290 (0.065)	<.001
MMEF, L/min	NA	−5.213 (0.980)	<.001	−4.522 (0.927)	<.001	−3.865 (0.916)	<.001
**COPD, odds ratio (95% CI)**							
NLR							
Continuous	765/7466	1.11 (1.05-1.17)^e^	<.001	1.10 (1.04-1.17)^e^	<.001	1.09 (1.03-1.15)^e^	.006
NLR category 1^f^							
Low	550/5973	1 [Reference]	NA	1 [Reference]	NA	1 [Reference]	NA
High	215/1943	1.53 (1.29-1.83)	<.001	1.56 (1.29-1.88)	<.001	1.49 (1.23-1.80)	<.001
NLR category 2^g^							
Low	410/4570	1 [Reference]	NA	1 [Reference]	NA	1 [Reference]	NA
High	355/2896	1.32 (1.13-1.54)	<.001	1.31 (1.11-1.55)	.001	1.27 (1.07-1.51)	.005

Abbreviations: COPD, chronic obstructive pulmonary disease; FEV_1_, forced respiratory volume in the first second; FEV_1_/FVC, percentage of vital capacity exhaled in the first second; FVC, forced vital capacity; NA, not applicable; NLR, neutrophil to lymphocyte ratio; MMEF, maximal mid-expiratory flow rate.

^a^ Mixed-effect linear model for lung function parameters and mixed-effect logistic model for COPD were used with random participant-specific intercepts to account for the correlation of repeated measures.

^b^ Model 1 adjusted for age, body mass index (categorized as underweight or normal weight, overweight, and obese), and height.

^c^ Model 2 additionally adjusted for smoking status (categorized as current, former, and never smoker), pack-years, alcohol consumption (categorized as abstainer, low, intermediate, and high), and education (categorized as ≤12 years, 13-16 years, and >16 years).

^d^ Model 3 (ie, the fully-adjusted model) additionally adjusted for hypertension, stroke, coronary heart disease, diabetes, and chronic lung conditions.

^e^ Calculated per 1-unit increase in NLR.

^f^ Category 1 used NLR greater than 3.00 as the cutoff value for high NLR.

^g^ Category 2 used NLR greater than 2.27 as the cutoff value for high NLR.

### Longitudinal Association of NLR With Lung Function

**Figure 3.**
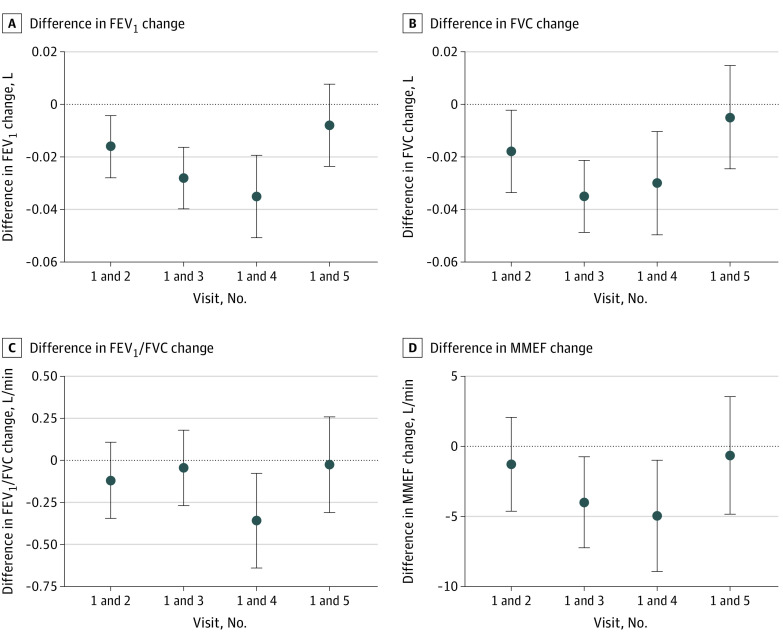
Associations of Change of Neutrophil to Lymphocyte Ratio Between the First and up to the Fifth Visit With Lung Function Change FEV_1_ indicates forced respiratory volume in the first second; FEV_1_/FVC, percentage of vital capacity exhaled in the first second; FVC, forced vital capacity; and MMEF, maximal mid-expiratory flow rate.

We assessed associations of the first visit NLR and rate of NLR change during follow-up of approximately 13 years with corresponding rate of lung function change. There were 1336 patients with at least 2 visits (mean [SD] follow-up, 3.7 [1.7] years), 1163 participants with at least 3 visits (mean [SD] follow-up, 6.8 [1.9] years), 998 participants with at least 4 visits (mean [SD] follow-up, 10.0 [2.0] years), and 777 participants with at least 5 visits (mean [SD] follow-up, 13.3 [2.5] years). The first visit NLR was negatively associated with the rate of lung function change up to the third visit (eTable 3 in the Supplement). Up to the fifth visit, NLR changes were negatively associated with lung function changes (Figure 3), but were only associated with the FEV_1_ and FVC changes up to the fourth visit (eTable 3 in the Supplement). In particular, a 1-unit increase in NLR change was associated with mean (SE) decreases of 0.019 (0.008) L in FEV_1_ and 0.032 (0.009) L in FVC between the first and second visits, and 0.035 (0.008) L in FEV_1_ and 0.030 (0.010) L in FVC first and fourth visits. Change rates of NLR showed similarly negative but much weaker associations with the change rates of lung function up to the fourth visit (eFigure in the Supplement).

### Association of NLR With COPD Incidence

Table 2 and Figure 2B show the associations of continuous NLR and 2 NLR categories with COPD incidence in 1411 participants without COPD at baseline. A 1-unit increase in the NLR was associated with a 27% higher risk of COPD in the crude model (hazard ratio [HR], 1.27 [95% CI, 1.05-1.51]; *P* = .01). Controlling for other potential covariates attenuated the association, but it was still statistically significant (HR, 1.22 [95% CI, 1.01-1.49]; *P* = .046). The high NLR group in category 1 was not associated with risk of COPD compared with the low NLR group (HR, 1.41 [95% CI, 0.75-2.64]; *P* = .28), nor was the high NLR group in category 2 (HR, 1.19 [95% CI, 0.79-1.79]; *P* = .41).

**Table 2.** Association of Neutrophil to Lymphocyte Ratio With COPD Incidence in Normative Aging Study

Indicator	Patients, No. with COPD/total No.	Model 1^a^		Model 2^b^		Model 3^c^	
		HR (95% CI)	*P* value	HR (95% CI)	*P* value	HR (95% CI)	*P* value
NLR	152/1411	1.27 (1.05-1.51)^e^	.01	1.23 (1.00-1.47)^e^	.049	1.22 (1.01-1.49)^e^	.046
Category 1 NLR status^e^							
Low	141/1320	1 [Reference]	NA	1 [Reference]	NA	1 [Reference]	NA
High	11/91	1.50 (0.81-2.77)	.20	1.40 (0.75-2.62)	.29	1.41 (0.75-2.64)	.28
Category 2 NLR status^f^							
Low	122/1163	1 [Reference]	NA	1 [Reference]	NA	1 [Reference]	NA
High	30/248	1.39 (0.94-2.08)	.10	1.20 (0.80-1.80)	.38	1.19 (0.79-1.79)	.41

Abbreviation: COPD, chronic obstructive pulmonary disease; HR, hazard ratio; NA, not applicable; NLR, neutrophil to lymphocyte ratio.

^a^ Model 1 adjusted for age, body mass index (categorized as underweight or normal weight, overweight, and obese), and height.

^b^ Model 2 additionally adjusted for smoking status (categorized as current, former, and never smoker), pack-years, alcohol consumption (categorized as abstainer, low, intermediate, and high), and education (categorized as ≤12 years, 13-16 years, and >16 years).

^c^ Model 3 (ie, the fully-adjusted model) additionally adjusted for hypertension, stroke, coronary heart disease, diabetes, and chronic lung conditions.

^d^ Calculated per 1-unit increase in NLR.

^e^ Category 1 used NLR greater than 3.00 as the cutoff value for high NLR.

^f^ Category 2 used NLR greater than 2.27 as the cutoff value for high NLR.

### Associations of NLR, cg05575921 Hypomethylation, and Lung Function

In the subgroup of visits with NLR and DNA methylation data, a 1-unit increase in NLR was associated with a mean (SE) decrease of 0.0048 (0.0021) in the methylation level of cg05575921 (*P* = .03) (eTable 4 in the Supplement). In subgroup analysis, the same increase in NLR yielded a mean (SE) decreases of 0.0066 (0.0030) in cg05575921 methylation levels for never smokers (*P* = .03) but the decrease of cg05575921 methylation levels in ever smokers was not statistically significant (−0.0046 [0.0029]; *P* = .12).

Both NLR and cg05575921 hypomethylation showed separate associations with lung function and COPD odds, and their corresponding estimates were essentially unchanged in the model adjusted for both biomarkers simultaneously (eTable 5 in the Supplement). We further performed subgroup analyses based on the NLR category 1 and a binary cg05575921 level (divided at the median value of 0.85). Steadily reduced lung function and steadily increased COPD odds were observed (eTable 6 in the Supplement). In category 1, participants with high NLRs (ie, ≥3.0) and low cg05575921 methylation (ie, <0.85) demonstrated the lowest lung function and the highest COPD odds. In particular, their odds were 4-fold higher (OR, 4.12 [95% CI, 2.02-8.37]) than those of participants with low NLRs (ie, <3.0) and high cg05575921 methylation (ie, ≥0.85).

Time-to-event analyses were also performed in the DNA methylation subgroup, which included 646 participants without COPD at the initial visit, among whom 14 participants developed COPD during a median (interquartile range) follow-up of approximately 4.0 (10.8) years (eTable 7 in the Supplement). A 1-unit increase in NLR was not associated with higher risk of COPD (HR, 1.04 [95% CI, 0.98-1.09]; *P* = .18), nor was a 1-SD decrease in cg05575921 (HR, 1.59 [95% CI, 0.91-2.80]; *P* = .10), which might be explained by relatively limited number of participants who developed COPD and short follow-up time.

Sensitivity analyses with additional adjustment of inverse probability weighting further validated the reliability of our main findings. Both primary analyses and the subgroup analyses yielded essentially unchanged estimates for the associations of NLR and cg05575921 with lung function, suggesting that results were not biased by the loss to follow-up and survivor bias.

### Mediation Analyses on NLR, cg05575921 Hypomethylation, and Lung Function

Finally, we explored whether the associations of NLR-related inflammation response with lung function were mediated by cg05575921 hypomethylation or whether the converse was plausible. eTable 8 in the Supplement presents the total association, natural direct association, natural indirect association, and proportion mediated by the mediator for the 2 scenarios. We fitted the exposure-mediator and mediator-outcome models simultaneously and found the association of NLR-related inflammation on lung function via cg05575921 hypomethylation was substantially mediated (mediation proportion range, 24.1%-40.7%). The mediated proportion of NLR-related inflammation on lung function via cg05575921 hypomethylation was essentially unchanged in ever smokers (mediation proportion range, 14.5%-47.6%) but strongly attenuated among never smokers (mediation proportion range, 1.2%-7.3%) (eTable 9 in the Supplement). In contrast, the association of cg05575921 hypomethylation with lung function was only slightly mediated by NLR (mediation proportion range, 0.7%-1.3%).

## Discussion

To our knowledge, this cohort study is the first study to investigate the associations of NLR with lung function impairment and COPD in a large longitudinal study. Furthermore, estimates of the associations of NLR with lung function change in longitudianl analyses were weaker than that in the initial analysis, which suggests that the association of age with lung function may be stronger than that of NLR. Using longitudinal data with repeated measurements of NLR and lung function obtained over 30 years, we found that NLR was associated with lung function and COPD as well as changes in lung function and COPD incidence during the study period. Additionally, NLR and cg05575921 methylation were associated with lung function impairment and COPD odds, and these factors could potentially be used to jointly classify the severity of the outcome. The adverse associations of NLR-related inflammation with lung function may be mediated by cg05575921 hypomethylation.

The association of NLR with lung function in the NAS population is consistent with prior reports on higher NLRs in patients with acute COPD exacerbations compared with their healthy counterparts or those with stable COPD.^9,10,24^ Several previous studies have analyzed the association of NLR with lung function, although a systematic review^10^ reported that most did not perform modeling analyses. A 2016 study^8^ of 479 Asian patients with COPD reported that a 1-unit increase in NLR was associated with a decrease of 0.035 L in FEV_1_. While this difference is larger than that found in our study, it could be attributed to the differences between a study population of only patients with COPD vs the general population used in this study. Furthermore, the longitudinal associations of NLR with lung function were weaker than that in the initial analysis of the association of NLR with lung function, which suggests that the association of lung function change with age may be stronger than the association of NLR. Our findings are epidemiologically relevant for providing evidence for the use of NLR in COPD prevention by providing critical information that may be ignored in clinical practice. After controlling for potential risk factors of COPD, including age, NLR remained associated with incident COPD, which may indicate that NLR is representative of the broad COPD risk status of the human body. Biologically, an increase of NLR is indicative of increases in neutrophils and decreases in lymphocytes in response to exogenous exposure. Increased neutrophils stimulate production of cytokines and subsequent destruction of lung tissue, possibly harming lung function and promoting COPD development.^25^ Although the associations of NLR with relevant respiratory health outcomes were examined in our study, owing to the limited statistical power, we are still far from establishing the optimal cutoff of NLR for the prediction of respiratory outcomes in the general population. Additionally, given the observational setting, the biological mechanisms underlying the causal association between NLR and COPD warrant further research.

Neutrophil to lymphocyte ratio may reflect the adverse respiratory associations of exogenous exposures with lung function. Our sensitivity analyses stratified by smoking status indicated that in ever smokers, NLR may provide incremental information for risk stratification. The sensitivity analyses also demonstrated that NLR was associated with lung function and COPD odds in never smokers, suggesting other nonsmoking exposures with respiratory outcomes (eg, air pollution^21,26,27^) may also alter NLR. Changes in NLR were also associated with lung function changes for up to approximately 10 years, suggesting NLR is a biomarker associated with long-term lung function changes in response to exogenous exposures. Public health professionals currently examine the outcomes of exogenous exposures on respiratory systems with direct measurements. These methods tend to be inaccurate owing to underreported use, recall bias (eg, tobacco smoking^28^), and bias caused by unmeasured residual confounding from ambient temperature and humidity (eg, air pollution^29^). Neutrophil to lymphocyte ratio might be a more straightforward and easily understood measurement of the outcomes of exogenous exposures on respiratory systems, but future studies are required to evaluate the use of NLR for this purpose.

Whole blood DNA methylation profiles are strongly associated with leukocyte distribution, as DNA is extracted from white blood cells with nucleus,^23^ and cg05575921 is strongly associated with smoking exposure.^11^ Therefore, we adjusted for leukocyte distribution estimated by Houseman algorithm, smoking status, and pack-years, and we still observed the association of NLR with cg05575921, which may suggest that NLR is associated with smoking-related COPD risk. Subgroup analysis based on smoking status showed that this association was slightly stronger in never smokers than in ever smokers, and NLR and cg05575921 were independently associated with lower lung function. These results indicate that NLR might capture non–smoking-related inflammation responses, which were not clearly captured by cg05575921 hypomethylation. The mediation of cg05575921 on the associations of NLR-related inflammation with lung function was essentially unchanged in ever smokers but strongly attenuated in never smokers. This suggests that cg05575921 methylation may mediate the association of NLR-related inflammation with respiratory outcomes associated with to smoking exposure, and other NLR-associated nonsmoking-related inflammation may impair lung function via other biological pathways.

In considering the value of NLR for COPD prevention, NLR is as useful as simple cell counts, such as C-reactive protein, interleukin 6, and tumor necrosis factor α,^6,7,9^ used at the clinical level to provide evidence of inflammation. Blood cell count estimation is a routine test more commonly requested in clinical practice than measuring serum inflammatory biomarkers. Clinicians may be able to use this information to quickly obtain the patients’ inflammatory status with NLR and thus decide on relevant interventions with medications or rehabilitation. Although DNA methylation measurement is generally too expensive for clinical practice, we found that tests integrating NLR with cg05575921 could characterize the extent of lung function impairment and COPD odds. This suggests that the use of both biomarkers may help clinicians identify individuals at a higher risk of developing COPD. Furthermore, DNA methylation can reliably estimate NLR in blood samples for which cell counts are not available, such as for cohorts established a few decades ago with samples in long term storage.^30^ This method may allow clinicians and epidemiologists to track historical and temporal changes in human immune profiles under exogenous exposures and corresponding respiratory diseases.

### Limitations

Our study has some limitations. First, NLR can differ drastically with underlying health. We adjusted for major chronic diseases in our models but cannot exclude residual confounding from other unknown health conditions. Second, healthier participants may be more likely to participate in subsequent examinations. Our sensitivity analyses with inverse probability weighting yielded similar estimates with the main findings, suggesting results were unbiased by follow-up loss. Third, we used the relatively conservative Global Initiative for Chronic Obstructive Lung Diseases stage II or higher standard for COPD, which may underestimate associations of NLR and cg05575921 with COPD odds and incidence, potentially attenuating associations in the subgroup analyses with fewer participants with COPD. The longitudinal association of NLR with FEV_1_ we observed may suggest that some of our participants may be very close to the threshold for diagnosis of COPD. Fourth, all selected participants were older white men, limiting the generalizability of our results, as NLR is sensitive to race and sex.^16^

## Conclusions

The findings of this cohort study suggest that NLR is a valuable, easily obtainable, and inexpensive biomarker associated with lung function impairment and COPD at the population level. Although DNA methylation is not yet used in routine clinical blood tests, we found that the assessment of DNA methylation along with NLR may help stratify the extent of lung function impairment and COPD progression at the population level. Hypomethylation of *AHRR* may also mediate the association of NLR-related inflammation with lung function. Multidisciplinary studies based on larger cohorts are required to confidently validate the utility of NLR and to explore the potential biological mechanisms involving *AHRR* (especially cg05575921).

## Notes

Supplement. **eAppendix.** Data Collection and DNA Methylation Data **eTable 1.** Characteristics of Participants From the Normative Aging Study for All Visits and the Subgroup With DNA Methylation Data **eTable 2.** Associations of Neutrophil to Lymphocyte Ratio With Lung Function and Odds of COPD Stratified by Smoking Status **eTable 3.** Associations of Neutrophil to Lymphocyte Ratio at First Visit and Change Rate Between First and Up to Fifth Visit, Including Change Rate of Lung Function in Subgroup With Available Data From 2 Visits **eTable 4.** Association of Neutrophil to Lymphocyte Ratio With cg05575921 **eTable 5.** Associations of Neutrophil to Lymphocyte Ratio and cg05575921 With Lung Function and Odds of COPD in the Subgroup With DNA Methylation Data **eTable 6.** Joint Associations of Neutrophil to Lymphocyte Ratio and cg05575921 With Lung Function and Odds of COPD in the Subgroup With DNA Methylation Data **eTable 7.** Association of Neutrophil to Lymphocyte Ratio With COPD Incidence in the Subgroup With DNA Methylation Data **eTable 8.** Estimates of Natural Direct and Natural Indirect Associations of NLR or cg05575921 on Lung Function and Proportion Mediated by cg05575921 or NLR **eTable 9.** Estimates of Natural Direct and Natural Indirect Associations of NLR or cg05575921 on Lung Function and Proportion Mediated by cg05575921 or NLR Stratified by Smoking Status **eFigure.** Associations of the Change Rate of Neutrophil to Lymphocyte Ratio Between the First and up to the Fifth Visit With Lung Function Change Rate
