# Supplementary material for: Association of Neutrophil to Lymphocyte Ratio With Pulmonary Function in a 30-Year Longitudinal Study of US Veterans
Source: JAMA Netw Open. 2020 Jul 13;3(7):e2010350. doi: 10.1001/jamanetworkopen.2020.10350 (PMC7358911; doi:10.1001/jamanetworkopen.2020.10350)
Supplement: Supplement. — eAppendix. Data Collection and DNA Methylation Data eTable 1. Characteristics of Participants From the Normative Aging Study for All Visits and the Subgroup With DNA Methylation Data eTable 2. Associations of Neutrophil to Lymphocyte Ratio With Lung Function and Odds of COPD Stratified by Smoking Status eTable 3. Associations of Neutrophil to Lymphocyte Ratio at First Visit and Change Rate Between First and Up to Fifth Visit, Including Change Rate of Lung Function in Subgroup With Available Data From 2 Visits eTable 4. Association of Neutrophil to Lymphocyte Ratio With cg05575921 eTable 5. Associations of Neutrophil to Lymphocyte Ratio and cg05575921 With Lung Function and Odds of COPD in the Subgroup With DNA Methylation Data eTable 6. Joint Associations of Neutrophil to Lymphocyte Ratio and cg05575921 With Lung Function and Odds of COPD in the Subgroup With DNA Methylation Data eTable 7. Association of Neutrophil to Lymphocyte Ratio With COPD Incidence in the Subgroup With DNA Methylation Data eTable 8. Estimates of Natural Direct and Natural Indirect Associations of NLR or cg05575921 on Lung Function and Proportion Mediated by cg05575921 or NLR eTable 9. Estimates of Natural Direct and Natural Indirect Associations of NLR or cg05575921 on Lung Function and Proportion Mediated by cg05575921 or NLR Stratified by Smoking Status eFigure. Associations of the Change Rate of Neutrophil to Lymphocyte Ratio Between the First and up to the Fifth Visit With Lung Function Change Rate [file jamanetwopen-3-e2010350-s001.pdf]

Supplementary Online Content

Gao X, Coull B, Lin X, et al. Association of neutrophil to lymphocyte ratio with pulmonary function in a 30-year longitudinal study of US veterans. *JAMA Netw Open*. 2020;3(7):2010350. doi:10.1001/jamanetworkopen.2020.10350

eAppendix. Data Collection and DNA Methylation Data
eTable 1. Characteristics of Participants From the Normative Aging Study for All Visits and the Subgroup With DNA Methylation Data
eTable 2. Associations of Neutrophil to Lymphocyte Ratio With Lung Function and Odds of COPD Stratified by Smoking Status
eTable 3. Associations of Neutrophil to Lymphocyte Ratio at First Visit and Change Rate Between First and Up to Fifth Visit, Including Change Rate of Lung Function in Subgroup With Available Data From 2 Visits
eTable 4. Association of Neutrophil to Lymphocyte Ratio With cg05575921
eTable 5. Associations of Neutrophil to Lymphocyte Ratio and cg05575921 With Lung Function and Odds of COPD in the Subgroup With DNA Methylation Data
eTable 6. Joint Associations of Neutrophil to Lymphocyte Ratio and cg05575921 With Lung Function and Odds of COPD in the Subgroup With DNA Methylation Data
eTable 7. Association of Neutrophil to Lymphocyte Ratio With COPD Incidence in the Subgroup With DNA Methylation Data
eTable 8. Estimates of Natural Direct and Natural Indirect Associations of NLR or cg05575921 on Lung Function and Proportion Mediated by cg05575921 or NLR
eTable 9. Estimates of Natural Direct and Natural Indirect Associations of NLR or cg05575921 on Lung Function and Proportion Mediated by cg05575921 or NLR Stratified by Smoking Status
eFigure. Associations of the Change Rate of Neutrophil to Lymphocyte Ratio Between the First and up to the Fifth Visit With Lung Function Change Rate

This supplementary material has been provided by the authors to give readers additional information about their work.

© 2020 Gao X et al. *JAMA Network Open.*

eAppendix. Data Collection and DNA Methylation Data

Data collection

As described [1], participants were asked to provide detailed information about their lifestyles, dietary habits, activity levels, and demographic factors at each of up to 13 visits between 1984 and 2018. BMIs (kg/m^2) were calculated from heights and weights. Major diseases including other chronic lung conditions (asthma, chronic bronchitis, and emphysema) were assessed based on medical histories and prior diagnoses. Spirometry was assessed in the standing position with a noseclip using a 10-liter water-filled survey-recording spirometer and an Eagle II minicomputer (Warren E. Collins, Braintree, Massachusetts). Standard methods were used to obtain FEV_1 (liters), FVC (liters), percent of vital capacity (FEV_1/FVC), and maximal mid-expiratory flow rate (MMEF, liters/minute, calculated as the average flow rate between 25% and 75% of FVC). All values were corrected to body temperature and pressure-saturated with water vapor. Spirometry may lead to over-diagnosis of COPD in the elderly [2]; thus, COPD was defined as meeting the Global Initiative for Chronic Obstructive Lung Diseases (GOLD) stage II (or higher) criteria (FEV_1/FVC <70% and FEV_1 <80% predicted) [3]. Smoking history, including status and pack-years, were assessed at the time of spirometry tests using the American Thoracic Society (ATS) questionnaire. NLR was calculated at each visit by dividing total absolute neutrophil counts by total absolute lymphocyte counts measured by automatic blood cell counters as a continuous variable. All participants were required to be fasting at the time blood was drawn. To explore potential cutoff points, two NLR values were defined as a low (reference) and high measurement: 1) a value of 3.0, a potential optimal cut-off for the upper limit of normal NLR [4]; and 2) a value of 2.27, the mean NLR of the selected participants.

© 2020 Gao X et al. *JAMA Network Open.*

DNA methylation data

As described [5], we used the QIAamp DNA Blood Kit (Qiagen, CA, USA) to extract DNA from buffy coat and performed bisulfite conversion with the EZ-96 DNA Methylation Kit (Zymo Research, CA, USA). We randomized chips across plates and randomized samples based on a two-stage age-stratified algorithm to minimize batch effects. We then measured DNA methylation profiles using the Illumina 450K BeadChip. After quality control, remaining samples were preprocessed using the Illumina-type background correction, dye-bias adjustment, and BMIQ normalization to generate methylation status. Methylation status of a specific CpG site was quantified as a β value ranging from 0 (no methylation) to 1 (full methylation). The methylation status of cg05575921 was retrieved from the whole epigenome data for this study.

References

1. Gao X, Coull B, Lin X, Vokonas P, Schwartz J, Baccarelli AA: **Nonsteroidal anti-inflammatory drugs modify the effect of short-term air pollution on lung function.** *Am J Respir Crit Care Med* 2019 (In press).

2. Qaseem A, Wilt TJ, Weinberger SE, Hanania NA, Criner G, van der Molen T, Marciniuk DD, Denberg T, Schunemann H, Wedzicha W, et al: **Diagnosis and management of stable chronic obstructive pulmonary disease: a clinical practice guideline update from the American College of Physicians, American College of Chest Physicians, American Thoracic Society, and European Respiratory Society.** *Ann Intern Med* 2011, **155:**179-191.

3. Vogelmeier CF, Criner GJ, Martinez FJ, Anzueto A, Barnes PJ, Bourbeau J, Celli BR, Chen R, Decramer M, Fabbri LM, et al: **Global Strategy for the Diagnosis,**

© 2020 Gao X et al. *JAMA Network Open.*

Management, and Prevention of Chronic Obstructive Lung Disease 2017 Report. GOLD Executive Summary. *Am J Respir Crit Care Med* 2017, **195:**557-582.

4. Vano YA, Oudard S, By MA, Tetu P, Thibault C, Aboudagga H, Scotte F, Elaidi R: **Optimal cut-off for neutrophil-to-lymphocyte ratio: Fact or Fantasy? A prospective cohort study in metastatic cancer patients.** *PLoS One* 2018, **13:**e0195042.

5. Gao X, Colicino E, Shen J, Kioumourtzoglou MA, Just AC, Nwanaji-Enwerem JC, Coull B, Lin X, Vokonas P, Zheng Y, et al: **Impacts of air pollution, temperature, and relative humidity on leukocyte distribution: An epigenetic perspective.** *Environ Int* 2019, **126:**395-405.

© 2020 Gao X et al. *JAMA Network Open*.

eTable 1. Characteristics of Participants From the Normative Aging Study for All Visits and the Subgroup With DNA Methylation Data

Characteristics	All visits (N = 7466)	Subset (N = 1228)
**Age [years]**	68.37 (9.37)	74.54 (7.08)
**FEV_1 [liters]**	2.67 (0.61)	2.53 (0.58)
**FVC [liters]**	3.56 (0.70)	3.39 (0.68)
**FEV_1/FVC [%]**	74.71 (7.48)	74.62 (7.59)
**MMEF [25%-75%, liters/min]**	256.28 (110.70)	234.20 (103.38)
**Neutrophil-to-lymphocyte ratio (NLR)**	2.27 (1.26)	2.82 (1.38)
**Smoking status**		
Current smoker	604 (8.1%)	42 (3.4%)
Former smoker	4546 (60.9%)	797 (64.9%)
Never smoker	2316 (31.0%)	389 (31.7%)
**Body mass index (BMI)** [b]		
Underweight or normal weight (<25.0)	1909 (25.6%)	281 (22.9%)
Overweight (≥25 to <30)	4015 (53.8%)	637 (51.8%)
Obese (≥30.0)	1541 (20.6%)	310 (25.2%)
**Alcohol consumption (2+ drinks per day)**	1563 (21.0%)	226 (18.4%)
**Years of education** [c]		
≤ 12 years	5534 (74.1%)	577 (47.0%)
13 – 16 years	773 (10.4%)	248 (20.2%)
> 16 years	1156 (15.5%)	403 (32.8%)
**Major diseases**		
Hypertension	4539 (60.8%)	908 (73.9%)
Stroke	366 (4.9%)	96 (7.8%)
Coronary heart disease (CHD)	1887 (25.3%)	398 (32.4%)
Diabetes	743 (10.0%)	180 (14.7%)
**Chronic obstructive pulmonary disease (COPD)**	765 (10.3%)	132 (10.8%)
**Other chronic lung conditions** [d]	897 (12.0%)	157 (12.8%)

a: Mean values (standard deviation) for continuous variables and n (%) for categorical variables
b: Data missing for 1 visit
c: Data missing for 3 visits
d: Including asthma, chronic bronchitis, and emphysema

© 2020 Gao X et al. *JAMA Network Open.*

eTable 2. Associations of Neutrophil to Lymphocyte Ratio With Lung Function and Odds of COPD Stratified by Smoking Status

<table>
<tr><th rowspan="2">Lung function parameters</th><th colspan="4">Never smokers [N = 2316] [b]</th><th colspan="4">Ever smokers [N = 5150] [c]</th></tr>
<tr><th colspan="2">Coefficients (SE)</th><th colspan="2">P-value</th><th colspan="2">Coefficients (SE)</th><th colspan="2">P-value</th></tr>
<tr><td>FEV$_1$ [liters]</td><td colspan="2">-0.017 (0.007)</td><td colspan="2">0.0272</td><td colspan="2">-0.026 (0.006)</td><td colspan="2"><0.0001</td></tr>
<tr><td>FVC [liters]</td><td colspan="2">-0.018 (0.009)</td><td colspan="2">0.0521</td><td colspan="2">-0.018 (0.006)</td><td colspan="2">0.0031</td></tr>
<tr><td>FEV$_1$/FVC [%]</td><td colspan="2">-0.110 (0.109)</td><td colspan="2">0.3163</td><td colspan="2">-0.397 (0.080)</td><td colspan="2"><0.0001</td></tr>
<tr><td>MMEF [25%-75%, liters/min]</td><td colspan="2">-2.682 (1.218)</td><td colspan="2">0.0424</td><td colspan="2">-5.005 (1.112)</td><td colspan="2"><0.0001</td></tr>
<tr><th>COPD</th><th>Number of cases</th><th colspan="2">Odds Ratio (95% CI)</th><th>P-value</th><th>Case number</th><th colspan="2">Odds Ratio (95% CI)</th><th>P-value</th></tr>
<tr><td>NLR [continuous]</td><td>83</td><td colspan="2">1.14 (0.98 – 1.32)</td><td>0.0888</td><td>682</td><td colspan="2">1.09 (1.03 – 1.16)</td><td>0.0058</td></tr>
<tr><td rowspan="2">NLR category 1
[Cutoff = 3.0]</td><td>Low [N = 1862, N_{case} = 58]</td><td colspan="3">Ref</td><td>Low [N = 4111, N_{case} = 492]</td><td colspan="3">Ref</td></tr>
<tr><td>High [N = 454, N_{case} = 25]</td><td colspan="2">1.39 (0.83 – 2.30)</td><td>0.2082</td><td>High [N = 1039, N_{case} = 190]</td><td colspan="2">1.60 (1.31 – 1.96)</td><td><0.0001</td></tr>
<tr><td rowspan="2">NLR category 2
[Cutoff = 2.27]</td><td>Low [N = 1415, N_{case} = 42]</td><td colspan="3">Ref</td><td>Low [N = 3155, N_{case} = 368]</td><td colspan="3">Ref</td></tr>
<tr><td>High [N = 901, N_{case} = 41]</td><td colspan="2">1.12 (0.70 – 1.78)</td><td>0.6466</td><td>High [N = 1995, N_{case} = 314]</td><td colspan="2">1.35 (1.12 – 1.62)</td><td>0.0011</td></tr>
</table>

a: Mixed-effect linear model for lung function parameters and mixed-effect logistic model for COPD were used with random participant-specific intercepts to account for the correlation of repeated measures

b: Model adjusted for age (years), body mass index (BMI, underweight or normal weight/ overweight/ obese), height (m), alcohol consumption (abstainer/ low/ intermediate/ high), education (≤12 years, 13-16 years, >16 years), hypertension, stroke, coronary heart disease (CHD), diabetes, and chronic lung conditions

c: Model additionally adjusted for pack-years

SE = Standard error; CIs = Confidence interval

© 2020 Gao X et al. *JAMA Network Open.*

eTable 3. Associations of Neutrophil to Lymphocyte Ratio at First Visit and Change Rate Between First and Up to Fifth Visit, Including Change Rate of Lung Function in Subgroup With Available Data From 2 Visits

**Visits**	**Average follow-up time (Year)**	**Predictor**	**Lung function parameters**	**Change between two visits (per unit)**		**Change rate between two visits (per unit/year)**	
				**Coefficients (SE)**	**P-value**	**Coefficients (SE)**	**P-value**
1st and 2nd visits [N = 1,336]	3.67	NLR at 1st visit [b]	FEV_1	-0.019 (0.008)	0.0105	-0.004 (0.002)	0.0471
			FVC	-0.032 (0.009)	0.0007	-0.008 (0.003)	0.0022
			FEV_1/FVC	-0.060 (0.139)	0.6667	-0.038 (0.042)	0.3592
			MMEF	-0.583 (2.079)	0.7793	-0.186 (0.604)	0.7574
		NLR change [c]	FEV_1	-0.016 (0.006)	0.0112		
			FVC	-0.018 (0.008)	0.0219		
			FEV_1/FVC	-0.119 (0.115)	0.3002		
			MMEF	-1.282 (1.710)	0.4535		
		NLR change rate [c]	FEV_1			-0.004 (0.006)	0.4604
			FVC			-0.0005 (0.007)	0.9481
			FEV_1/FVC			-0.113 (0.114)	0.3225
			MMEF			-1.178 (1.647)	0.4746
1st and 3rd visits [N = 1,163]	6.82	NLR at 1st visit [b]	FEV_1	-0.008 (0.010)	0.4073	-0.0004 (0.001)	0.7924
			FVC	-0.023 (0.012)	0.0502	-0.003 (0.002)	0.1310
			FEV_1/FVC	-0.187 (0.176)	0.2889	-0.028 (0.026)	0.2922
			MMEF	-2.027 (2.564)	0.4293	-0.447 (0.381)	0.2400
		NLR change [c]	FEV_1	-0.028 (0.006)	<0.0001		
			FVC	-0.035 (0.007)	<0.0001		
			FEV_1/FVC	-0.044 (0.114)	0.6997		
			MMEF	-3.992 (1.658)	0.0162		
		NLR change rate [c]	FEV_1			-0.013 (0.006)	0.0348
			FVC			-0.019 (0.007)	0.0079
			FEV_1/FVC			-0.056 (0.114)	0.6214
			MMEF			-1.103 (1.649)	0.5307

© 2020 Gao X et al. *JAMA Network Open.*

Visits	Average follow-up time (Year)	Predictor	Lung function parameters	Change between two visits (per unit)		Change rate between two visits (per unit/year)	
				Coefficients (SE)	P-value	Coefficients (SE)	P-value
1st and 4th visits [N = 988]	9.95	NLR at 1st visit [b]	FEV_1	0.003 (0.012)	0.7932	0.001 (0.001)	0.3139
			FVC	-0.005 (0.014)	0.7042	-0.0005 (0.001)	0.7346
			FEV_1/FVC	0.184 (0.213)	0.3891	0.019 (0.021)	0.3763
			MMEF	1.021 (0.299)	0.7327	0.222 (0.306)	0.4685
		NLR change [c]	FEV_1	-0.035 (0.008)	<0.0001		
			FVC	-0.030 (0.010)	0.0015		
			FEV_1/FVC	-0.358 (0.143)	0.0125		
			MMEF	-4.966 (2.021)	0.0142		
		NLR change rate [c]	FEV_1			-0.014 (0.008)	0.0892
			FVC			-0.005 (0.010)	0.5822
			FEV_1/FVC			-0.289 (0.149)	0.0519
			MMEF			-1.794 (2.141)	0.4023
1st and 5th visits [N = 777]	13.33	NLR at 1st visit [b]	FEV_1	0.002 (0.015)	0.9160	0.0006 (0.001)	0.6098
			FVC	0.009 (0.018)	0.6145	0.001 (0.001)	0.2879
			FEV_1/FVC	-0.235 (0.261)	0.3672	-0.020 (0.020)	0.3232
			MMEF	0.682 (3.829)	0.8586	0.068 (0.288)	0.8150
		NLR change [c]	FEV_1	-0.008 (0.008)	0.3241		
			FVC	-0.005 (0.010)	0.6212		
			FEV_1/FVC	-0.026 (0.145)	0.8570		
			MMEF	-0.664 (2.140)	0.7565		
		NLR change rate [c]	FEV_1			0.0002 (0.008)	0.9826
			FVC			0.004 (0.010)	0.6936
			FEV_1/FVC			0.020 (0.145)	0.8933
			MMEF			1.562 (2.129)	0.4632

a: Change = Measures of last visit – Measures of 1st visit; Change rate = Change / Duration between the two visits (year)
b: Models adjusted for the covariates collected at the 1st visit, including: age (years), body mass index (BMI, underweight or normal weight/ overweight/ obese), height, smoking status (current/ former/ never), pack-years, alcohol intake (<2 drinks per day or ≥2 drinks per day), education (≤12 years, 13-16 years, >16 years), hypertension, stroke, coronary heart disease, diabetes, chronic lung conditions, and the corresponding lung function parameters measured at the 1st visit
c: Models additionally adjusted for NLR measured at the 1st visit

© 2020 Gao X et al. *JAMA Network Open.*

eTable 4. Association of Neutrophil to Lymphocyte Ratio With cg05575921

**Sample**	**Model**	
	**Coefficients (SE)**	**P-value**
All visits from the subset [N = 1228] [a]	-0.0048 (0.0021)	0.026
Subgroup [b]		
Never smokers [N = 389]	-0.0066 (0.0030)	0.028
Ever smokers [N = 839]	-0.0046 (0.0029)	0.119

a: Model adjusted for age (years), body mass index (BMI, underweight or normal weight/ overweight/ obese), height (m), smoking status (current/ former/ never smoker), pack-years, alcohol consumption (abstainer/ low/ intermediate/ high), education (≤12 years, 13-16 years, >16 years), hypertension, stroke, coronary heart disease (CHD), diabetes, chronic lung conditions, the leukocyte distribution estimated by Houseman's algorithm, and the random batch effect

b: Model adjusted for all aforementioned covariates except smoking status. Pack-years was only controlled for in the subgroup of ever smokers
SE = Standard error

© 2020 Gao X et al. *JAMA Network Open.*

eTable 5. Associations of Neutrophil to Lymphocyte Ratio and cg05575921 With Lung Function and Odds of COPD in the Subgroup With DNA Methylation Data

a) **Individual model** [a]

Biomarkers	NLR [per one unit increase]		cg05575921 [per SD decrease]	
**Lung function parameters**	**Coefficients (SE)**	**P-value**	**Coefficients (SE)**	**P-value**
FEV_1 [liters]	-0.032 (0.009)	0.0005	-0.108 (0.016)	<0.0001
FVC [liters]	-0.028 (0.011)	0.009	-0.071 (0.019)	0.0002
FEV_1/FVC [%]	-0.320 (0.137)	0.020	-1.749 (0.241)	<0.0001
MMEF [25%-75%, liters/min]	-4.641 (1.899)	0.015	-22.717 (3.175)	<0.0001
**COPD [N_{case} = 132]**	**Odds Ratio (95% CI)**	**P-value**	**Odds Ratio (95% CI)**	**P-value**
	1.13 (1.00 – 1.29)	0.049	1.74 (1.40 – 2.15)	<0.0001

b) **Mutually adjusted model** [b]

Biomarkers	NLR [per one unit increase]		cg05575921 [per SD decrease]	
**Lung function parameters**	**Coefficients (SE)**	**P-value**	**Coefficients (SE)**	**P-value**
FEV_1 [liters]	-0.030 (0.009)	0.001	-0.104 (0.016)	<0.0001
FVC [liters]	-0.026 (0.011)	0.015	-0.068 (0.019)	0.0004
FEV_1/FVC [%]	-0.300 (0.135)	0.026	-1.680 (0.240)	<0.0001
MMEF [25%-75%, liters/min]	-4.587 (1.781)	0.010	-21.744 (3.165)	<0.0001
**COPD [N_{case} = 132]**	**Odds Ratio (95% CI)**	**P-value**	**Odds Ratio (95% CI)**	**P-value**
	1.11 (0.98 – 1.25)	0.098	1.67 (1.36 – 2.05)	<0.0001

a: Mixed-effect linear model for lung function parameters and mixed-effect logistic model for COPD were used with random participant-specific intercepts to account for the correlation of repeated measures. Models adjusted for age (years), body mass index (BMI, underweight or normal weight/ overweight/ obese), height, smoking status (current/ former/ never), pack-years, alcohol intake (<2 drinks per day or ≥2 drinks per day), education, hypertension, stroke, coronary heart disease, diabetes, and chronic lung conditions. For models with cg05575921, the leukocyte distribution estimated by Houseman's algorithm, and the random batch effect were additionally adjusted

b: This model adjusted for both biomarkers and all the covariates mentioned above except the cell composition estimated by Houseman's algorithm because the NLR is very highly associated with the leucocyte distribution

© 2020 Gao X et al. *JAMA Network Open.*

eTable 6. Joint Associations of Neutrophil to Lymphocyte Ratio and cg05575921 With Lung Function and Odds of COPD in the Subgroup With DNA Methylation Data[a]

**Lung function**			
**Category [N]**	**Parameter**	**Coefficients (SE)**	**P-value**
NLR <3 & cg05575921 ≥0.85 [N = 402]	FEV_1 [liters]	Ref	
NLR ≥3 & cg05575921 ≥0.85 [N = 212]		-0.065 (0.035)	0.064
NLR <3 & cg05575921 <0.85 [N = 398]		-0.095 (0.039)	0.015
NLR ≥3 & cg05575921 <0.85 [N = 216]		-0.198 (0.041)	<0.0001
NLR <3 & cg05575921 ≥0.85 [N = 402]	FVC [liters]	Ref	
NLR ≥3 & cg05575921 ≥0.85 [N = 212]		-0.011 (0.041)	0.793
NLR <3 & cg05575921 <0.85 [N = 398]		-0.079 (0.045)	0.079
NLR ≥3 & cg05575921 <0.85 [N = 216]		-0.132 (0.048)	0.006
NLR <3 & cg05575921 ≥0.85 [N = 402]	FEV_1/FVC [%]	Ref	
NLR ≥3 & cg05575921 ≥0.85 [N = 212]		-0.784 (0.580)	0.177
NLR <3 & cg05575921 <0.85 [N = 398]		-1.921 (0.521)	0.0002
NLR ≥3 & cg05575921 <0.85 [N = 216]		-3.169 (0.610)	<0.0001
NLR <3 & cg05575921 ≥0.85 [N = 402]	MMEF [25%-75%, liters/min]	Ref	
NLR ≥3 & cg05575921 ≥0.85 [N = 212]		-14.304 (7.520)	0.991
NLR <3 & cg05575921 <0.85 [N = 398]		-17.022 (6.856)	0.233
NLR ≥3 & cg05575921 <0.85 [N = 216]		-35.784 (7.925)	0.002
**COPD**			
**Category [b]**	N_{case}	**Odds Ratio (95% CI)**	**P-value**
NLR <3 & cg05575921 ≥0.85 [N = 402]	12	Ref	
NLR ≥3 & cg05575921 ≥0.85 [N = 212]	12	2.13 (0.91 – 4.95)	0.080
NLR <3 & cg05575921 <0.85 [N = 398]	63	3.17 (1.60 – 6.25)	0.0009
NLR ≥3 & cg05575921 <0.85 [N = 216]	45	4.12 (2.02 – 8.37)	<0.0001

a: Mixed-effect linear model for lung function parameters and mixed-effect logistic model for COPD were used with random participant-specific intercepts to account for the correlation of repeated measures. Models adjusted for age (years), body mass index (BMI, underweight or normal weight/ overweight/ obese), height, smoking status (current/ former/ never), pack-years, alcohol intake (< 2 drinks per day or ≥ 2 drinks per day), education, hypertension, stroke, coronary heart disease, diabetes, chronic lung conditions, and the random batch effect for DNA methylation measurement

© 2020 Gao X et al. *JAMA Network Open.*

eTable 7. Association of Neutrophil to Lymphocyte Ratio With COPD Incidence in the Subgroup With DNA Methylation Data

**Indicator**	**Model [a]**	
	**HR (95% CI)**	**P-value**
NLR [per one unit increase]	1.04 (0.98 – 1.09)	0.180
cg05575921 [per one SD decrease]	1.59 (0.91 – 2.80)	0.104

a: Model adjusted for age (years), body mass index (BMI, underweight or normal weight/ overweight/ obese), height (m), smoking status (current/ former/ never smoker), pack-years, alcohol consumption (abstainer/ low/ intermediate/ high), education (≤12 years, 13-16 years, >16 years), hypertension, stroke, coronary heart disease (CHD), diabetes, and chronic lung conditions. For models with cg05575921, the leukocyte distribution estimated by Houseman's algorithm and the random batch effect were additionally adjusted

HR = Hazard ratio; CI = Confidence interval; SD = Standard error

© 2020 Gao X et al. *JAMA Network Open.*

eTable 8. Estimates of Natural Direct and Natural Indirect Associations of NLR or cg05575921 on Lung Function and Proportion Mediated by cg05575921 or NLR

a) Effects of NLR on lung function via cg05575921 methylation

**Lung function parameters**	**Total effect (SE)**	**Natural direct effect (SE)**	**Natural indirect effect (SE)**	**Proportion mediated**
FEV_1 [liters]	-0.0283 (0.0155)	-0.0209 (0.0153)	-0.0074 (0.0033)	26.2%
FVC [liters]	-0.0123 (0.0172)	-0.0073 (0.0181)	-0.0050 (0.0025)	40.7%
FEV_1/FVC [%]	-0.4886 (0.2325)	-0.3708 (0.2276)	-0.1178 (0.0521)	24.1%
MMEF [25%-75%, liters/min]	-5.7951 (3.0617)	-4.3600 (3.0078)	-1.4351 (0.6395)	24.8%

b) Effects of cg05575921 methylation on lung function via NLR

**Lung function parameters**	**Total effect (SE)**	**Natural direct effect (SE)**	**Natural indirect effect (SE)**	**Proportion mediated**
FEV_1 [liters]	-0.1156 (0.0154)	-0.1143 (0.0154)	-0.0013 (0.0011)	1.1%
FVC [liters]	-0.0791 (0.0182)	-0.0786 (0.0182)	-0.0005 (0.0012)	0.7%
FEV_1/FVC [%]	-1.7942 (0.2302)	-1.7740 (0.2305)	-0.0201 (0.0170)	1.1%
MMEF [25%-75%, liters/min]	-22.6068 (3.0214)	-22.3235 (3.0248)	-0.2833 (0.2282)	1.3%

a: Effects were shown by one-unit increase in NLR and one-SD decrease in cg05575921. Models adjusted for age (years), body mass index (BMI, underweight or normal weight/ overweight/ obese), height, smoking status (current/ former/ never), pack-years, alcohol intake (<2 drinks per day or ≥2 drinks per day), education, hypertension, stroke, coronary heart disease, diabetes, chronic lung condition, the leukocyte distribution estimated by Houseman's algorithm, and the batch number of DNA methylation measurements

© 2020 Gao X et al. *JAMA Network Open.*

eTable 9. Estimates of Natural Direct and Natural Indirect Associations of NLR or cg05575921 on Lung Function and Proportion Mediated by cg05575921 or NLR Stratified by Smoking Status[a]

1) Never smoker [N = 389]

c) Effects of NLR on lung function via cg05575921 methylation

Lung function parameters	Total effect (SE)	Natural direct effect (SE)	Natural indirect effect (SE)	Proportion mediated
FEV_1 [liters]	-0.0629 (0.0251)	-0.0583 (0.0252)	-0.0046 (0.0036)	7.3%
FVC [liters]	-0.0715 (0.0315)	-0.0706 (0.0318)	-0.0009 (0.0039)	1.2%
FEV_1/FVC [%]	-0.1706 (0.0860)	-0.1637 (0.4239)	-0.0069 (0.4261)	4.0%
MMEF [25%-75%, liters/min]	-3.2124 (3.3652)	-3.1424 (3.3715)	-0.0700 (0.2202)	2.2%

d) Effects of cg05575921 methylation on lung function via NLR

Lung function parameters	Total effect (SE)	Natural direct effect (SE)	Natural indirect effect (SE)	Proportion mediated
FEV_1 [liters]	-0.0543 (0.0357)	-0.0558 (0.0354)	0.0015 (0.0043)	-2.7%
FVC [liters]	-0.0135 (0.0448)	-0.0153 (0.0446)	0.0018 (0.0052)	-13.4%
FEV_1/FVC [%]	-1.8514 (0.5820)	-1.8531 (0.5820)	0.0017 (0.0113)	-0.1%
MMEF [25%-75%, liters/min]	-9.4291 (7.9660)	-9.6072 (7.9509)	0.1782 (0.5288)	-1.9%

© 2020 Gao X et al. *JAMA Network Open.*

2) Ever smoker [N = 839]

e) Effects of NLR on lung function via cg05575921 methylation

Lung function parameters	Total effect (SE)	Natural direct effect (SE)	Natural indirect effect (SE)	Proportion mediated
FEV_1 [liters]	-0.0146 (0.0182)	-0.0077 (0.0177)	-0.0070 (0.0044)	47.6%
FVC [liters]	-0.0147 (0.0205)	-0.0098 (0.0206)	-0.0049 (0.0032)	33.3%
FEV_1/FVC [%]	-0.7592 (0.2758)	-0.6492 (0.2680)	-0.1100 (0.0683)	14.5%
MMEF [25%-75%, liters/min]	-5.3239 (3.6065)	-3.8876 (3.5060)	-1.4363 (0.8914)	26.9%

f) Effects of cg05575921 methylation on lung function via NLR

Lung function parameters	Total effect (SE)	Natural direct effect (SE)	Natural indirect effect (SE)	Proportion mediated
FEV_1 [liters]	-0.1246 (0.0175)	-0.1241 (0.0175)	-0.0004 (0.0010)	0.3%
FVC [liters]	-0.0866 (0.0202)	-0.0858 (0.0202)	-0.0008 (0.0012)	0.9%
FEV_1/FVC [%]	-1.8459 (0.2539)	-1.8176 (0.2537)	-0.0283 (0.0223)	1.5%
MMEF [25%-75%, liters/min]	-24.9501 (3.2796)	-24.7589 (3.2826)	-0.1913 (0.2163)	0.8%

a: The effects were shown by one-unit increase in NLR and one-SD decrease in cg05575921. Models adjusted for age (years), body mass index (BMI, underweight or normal weight/ overweight/ obese), height, alcohol intake (<2 drinks per day or ≥2 drinks per day), education, hypertension, stroke, coronary heart disease, diabetes, chronic lung condition, the leukocyte distribution estimated by Houseman's algorithm, and the batch number of DNA methylation measurements. Pack-years was additionally adjusted for in the model for ever smokers

© 2020 Gao X et al. *JAMA Network Open.*

eFigure. Associations of the Change Rate of Neutrophil to Lymphocyte Ratio Between the First and up to the Fifth Visit With Lung Function Change Rate

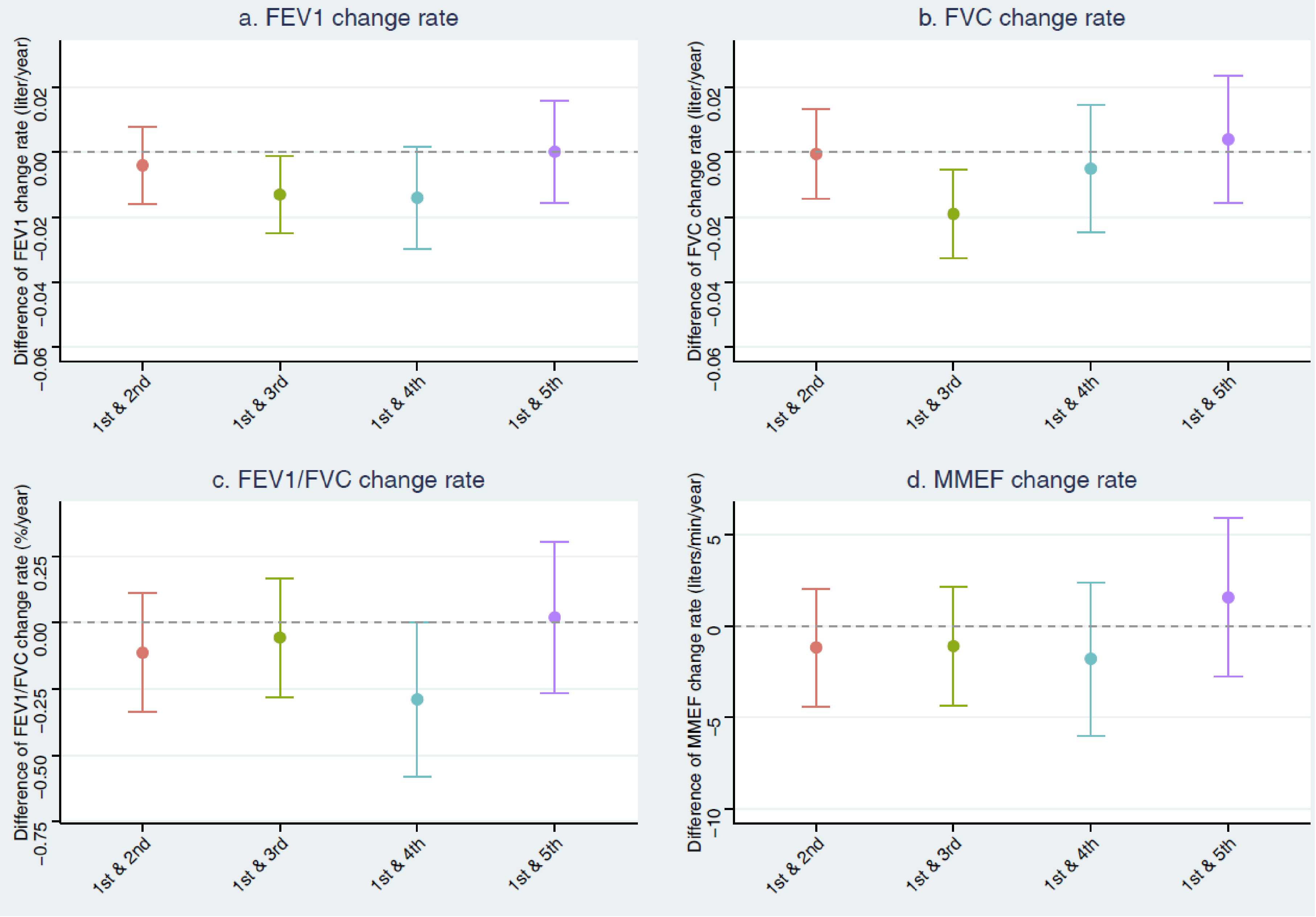

Figure S1 Associations of the change rate of neutrophil−to−lymphocyte ratio between 1st and up to 5th visit with lung function change rate

© 2020 Gao X et al. *JAMA Network Open.*
